# Association between vitamin D status, physical performance, sex, and lifestyle factors: a cross-sectional study of community-dwelling Kosovar adults aged 40 years and older

**DOI:** 10.1007/s00394-023-03303-9

**Published:** 2024-01-09

**Authors:** Ermira Krasniqi, Arben Boshnjaku, Antigona Ukëhaxhaj, Karl-Heinz Wagner, Barbara Wessner

**Affiliations:** 1 Research Platform Active Ageing, University of Vienna, Josef-Holaubek-Platz 2, 1090 Vienna, Austria; 2 Vienna Doctoral School of Pharmaceutical, Nutritional and Sport Sciences (PhaNuSpo), University of Vienna, Josef-Holaubek-Platz 2, 1090 Vienna, Austria; 3 Centre for Sport Science and University Sports, University of Vienna, Auf Der Schmelz 6, 1150 Vienna, Austria; 4 Faculty of Life Sciences, Department of Nutritional Sciences, University of Vienna, Josef-Holaubek-Platz 2, 1090 Vienna, Austria; 5 University “Fehmi Agani” in Gjakova, Ismail Qemali N.N. 50000, Gjakovë, Kosovo; 6 National Institute of Public Health of Kosovo, Centre for Laboratory Testing, Mother Teresa, n.n., Hospital District, 10000 Prishtina, Kosovo

**Keywords:** Vitamin D, 25-Hydroxyvitamin D, Physical performance, Muscle strength, Physical activity

## Abstract

**Purpose:**

Vitamin D status and its association with age-related decline in physical performance and strength have already been highlighted in various ways, but data on the situation in developing countries are scarce. This study aimed to investigate vitamin D status, its association with muscle mass and function, and other potential determinants such as age, sex, lifestyle factors (physical activity, dietary behavior), self-perceived health status, medication intake, education and financial situation in adults from Kosovo.

**Methods:**

This cross-sectional study included 297 participants (54.5% women), aged ≥ 40 years. Serum 25-hydroxyvitamin D (25(OH)D) concentration, hand grip strength and physical performance tests, body composition, vitamin D dietary intake and knowledge were assessed. The interaction between serum 25(OH)D status, lifestyle factors and muscle traits was investigated.

**Results:**

Vitamin D deficiency (< 50 nmol/L) was observed in 47.5% of the total population, of whom 14.7% of them were severely deficient (< 30 nmol/L). No associations were found between 25(OH)D concentration and age. Daily dietary intake of vitamin D was low (1.89 ± 0.67 µg) and 87.6% of individuals did not take vitamin D supplements. However, vitamin D supplementation was the only variable that added statistical significance (*p* < 0.05) to the prediction of vitamin D status (3.8%). On the other hand, age, medication intake and vitamin D level contributed significantly to the overall regression model, explaining 24.9% of the 30-s chair stand performance as an indicator of lower-body strength endurance.

**Conclusion:**

Vitamin D deficiency is highly prevalent among community-dwelling adults in Kosovo and low serum 25(OH)D has been associated with low muscle strength. This implies an urgent need for the development of comprehensive prevention strategies, focusing on pharmacological (supplementation) but also on non-pharmacological strategies such as education, food fortification or lifestyle advices.

**Supplementary Information:**

The online version contains supplementary material available at 10.1007/s00394-023-03303-9.

## Introduction

Vitamin D is a topic of global interest to the scientific and clinical communities as well as the general public. Vitamin D deficiency has often been described as a growing concern for people of all ages, particularly following evidence of its potential health effects [1]. Low levels of vitamin D have been reported worldwide leading to the classification of vitamin D deficiency as a pandemic [2].

Fat-soluble vitamin D includes ergocalciferol (D_2_) and cholecalciferol (D_3_). Both forms can be obtained from either food or supplements, although vitamin D3 is mainly produced in the skin from 7-dehydrocholesterol under ultraviolet B radiation. After entering the bloodstream, vitamin D is converted in the liver to 25-hydroxyvitamin D (the circulating form) and then in the kidneys to 1,25-dihydroxyvitamin D [1,25(OH)_2_D] (the biologically active form). Both forms circulate, bound to vitamin D-binding protein, whereas 1,25(OH)_2_D exerts its biological effect by binding to the intracellular vitamin D receptor (VDR) [1, 3, 4].

Nevertheless, the importance of vitamin D levels for general health and its role in skeletal muscle metabolism have been highlighted in several ways [5]. Initially, it was reported that inhibition of 1,25(OH)D binding to the VDR may also inhibit dependent mechanisms that regulate intracellular calcium entry [6]. VDR is expressed in the nucleus of human muscle cells, and its number is thought to decrease with age, thereby affecting muscle strength with ageing [7]. It has also been suggested that vitamin D may be included as a potential biomarker of the ageing process, although more studies are still needed in this area [8]. However, despite several studies supporting an association of vitamin D level with muscle strength and physical performance [5, 9, 10], the current state of knowledge remains inconclusive [11].

To date, serum measurement of the circulating form of 25(OH)D is considered the best biomarker for assessing vitamin D status in clinical practice. Despite the existing discrepancies regarding the optimal vitamin D level, it is generally recommended to be between 50 and 125 nmol/L (20–50 ng/mL) [12]. This can be achieved through sun exposure, dietary sources of vitamin D such as oily fish, eggs, meat, and some vitamin D fortified food products [13]. However, other factors such as age, skin pigmentation, clothing style, sunscreen use, air pollution, latitude and finally genetic profile may be important contributors to vitamin D deficiency [1, 4, 14], possibly impacting the wide range of vitamin D deficiency data in different countries.

From an epidemiological point of view, vitamin D deficiency is considered a public health problem, especially in older adults [13]. Although data on vitamin D status, its association with risk factors, health outcomes, and strategies to improve vitamin D status have been collected in developed countries, the same cannot be said for many developing countries [15]. To the best of our knowledge,  there are still no published data examining vitamin D status in the adult population of Kosovo. A similar lack of data can be observed in most neighbouring countries, but also in southern European countries [16].

Considering these arguments, the aim of this study was to evaluate vitamin D status, and its association with muscle mass and function (strength and performance) in community-dwelling adults from Kosovo. We also aimed to examine some possible determinants of vitamin D levels, such as demographics, biological sex, lifestyle, physical activity, dietary factors and self-perceived health status. We hypothesize that vitamin D deficiency  in this population is associated with poor muscle characteristics, nutritional status, low physical activity, and lower education and socioeconomic (financial situation) status in this population.

## Methods

### Study design, subjects and data collection

This cross-sectional study was conducted from December 1, 2019 until the February 23, 2020. All participants, were community-dwelling middle-aged and older men and women living in Kosovo, coming from two larger regions of Kosovo (out of a total of seven) Prishtina and Gjakova (total population 502,038 and 158,903 inhabitants, respectively). Inclusion criteria for this convenience sample included male and female participants over 40 years of age, with no upper limit; living in the community; coming from either the Prishtina or Gjakova region. Exclusion criteria were any acute illness that would prevent the participant from participating in any exercise test, and  any serious chronic illness that required continuous medical care. The recruitment process included recall of participants from a previous cross-sectional, observational study [17, 18] and advertisements for additional participants (throughout the city and older people’s association).

The data collection process was structured and carried out in a consistent manner throughout the study period, using the same test order and time by the same research group. The measurements were performed at the Laboratory for Human Biomarkers and the Sports Hall at the University “Fehmi Agani” in Gjakova (Kosovo), starting with blood sampling, and anthropometric and body composition measurements. After a break of about one hour with a light standardised meal, the physical performance tests were performed. Participants’ personal data on self-perceived health status, comorbidities, medications, socioeconomic status, nutritional status, vitamin D knowledge, physical activity, and sedentary behaviour, were collected by two health professionals.

### Serum/plasma collection and vitamin D status

Blood samples were collected in the morning after an overnight fast by licensed healthcare professionals. Blood samples were centrifuged (at 2.000 x g for 10 min at room temperature), and serum samples were initially stored at  – 20 °C and then shipped frozen in bulk to the Laboratory for Molecular Exercise Physiology, Centre for Sport Sciences and University Sports, University of Vienna (Austria) for vitamin D measurements. Total serum 25-(OH)D (including 25-OH vitamin D_2_ and 25-OH vitamin D_3_) was measured by enzyme-linked immunosorbent assay (ELISA) using a commercially available kit (EUROIMMUN Medizinische Labordiagnostika AG, Lübeck, Germany) according to the manufacturer’s instructions [19]. This was compared with other assays and showed high correlations (HPLC, *r*^2^ = 0.91, *n* = 80; LC–MS/MS, *r*^2^ = 0.93, *n* = 100; IDS 25-OH Vitamin D Direct EIA, *r*^2^ = 0.93, *n* = 231). The intra-assay coefficients of variation (CVs) were obtained by performing 40 measurements on each serum sample, while the inter-assay CVs were obtained by performing four measurements on ten different test runs, resulting in percentages of 5.22% and 7.82%, respectively. The ELISA test kit designed to use a competitive ELISA technique with a selected monoclonal antibody that identifies vitamin D. In this case, an unknown amount of 25-OH vitamin D from the participant's serum competes with biotin-labelled 25-OH vitamin D for the antibody binding sites in the microplate wells plate, while the calibrators/controls and samples were diluted 1:26 in working strength biotin. After a chromogen/substrate reaction, the colour intensity (which is inversely proportional to the serum 25-OH vitamin D concentration) was measured photometrically (PerkinElmer, Inc., Waltham, Massachusetts, United States) at a wavelength of 450 nm. MyAssays.com was used to generate a four-parameter logistic curve fit to calculate the vitamin D concentration in the samples. Among the discrepancies between cut-offs, it is generally accepted that the appropriate target for serum 25(OH)D concentration is 50 nmol/L, which we used as the cut-off between sufficiency and deficiency [1].

### Anthropometry, body composition and clinical characteristics

For anthropometric measurements the International Standards for Anthropometric Assessments [20] were followed, with participants being barefoot, wearing light indoor clotheing, after an overnight fast and without alcohol intake for the previous 12 h. Height was measured to the nearest 0.5 cm using a portable stadiometer (Seca, Hamburg, Germany) according to the stretch stature method, while body mass was measured to the nearest 0.1 kg using a digital scale (Seca, Hamburg, Germany).  Body mass index (BMI) was determined as body mass divided by the body height squared (expressed in kg/m^2^).

Segmental multifrequency tetrapolar bioelectric impedance analysis (BIA) at four frequencies (5, 50, 100 and 200 kHz) was used as the non-invasive method of choice to assess body composition (BodyStat Quadscan 4000, Isle of Man, United Kingdom). Measurements were performed according to the manufacturer’s recommendations and previously reported methods for the tetrapolar BIA [21], with the subjects comfortably supine with arms and legs abducted, no metal objects in clothing, and with two electrodes placed on each hand/foot on the same side. Each morning before the measurements, the device was calibrated using the manufacturer’s calibrator, which measures impedance at all frequencies.

### Isometric strength and physical performance

Isometric handgrip strength (HGS) of the sitting participant’s dominant hand was assessed using an adaptable dynamometer (JAMAR, Petterson Medical, Saint Paul, MN, USA) on two identical trials (the better one was recorded) [22].

Lower and upper body strength endurance were assessed by the 30-s chair-stand (CS) and 30-s arm-curl (AC) tests, respectively. The final outcome measures were the maximum number of repetitions of standing up and sitting down from a 46 cm high armless chair [23], and the maximum number of repetitions of dumbbell lifts (8 and 5 pounds for men and women, respectively) with the dominant hand in a seated position within 30 s [23]. In both cases, the testers stood beside the participant holding a stopwatch, signalled the start and the end of the test time, and counted the repetitions (the last repetition was counted if more than 50% of the range had been completed) [23].

Functional mobility was assessed using the timed up-and-go test (TUG) [23], in which the tester, standing sideways and counting time, signalled participants to stand up from an armless chair and walk as fast as possible without running to a cone 3 m away, turn around and sit back down in the chair.

Participants’ usual and fast walking speeds were measured by a gait speed test performed within 6 m of a 10 m course [22, 24]. The examiner stood next to the participant holding a stopwatch and signalled the start of the test while counting the time between the 2nd and 8th metre (leaving 2 metres in each direction for acceleration and deceleration). The better results of each of the two trials (for both speeds) were taken for further evaluation.

Aerobic endurance was assessed by a 6-min walking test (6MWT) [23, 24], in which participants were instructed to walk as far as possible at a self-selected pace within a 20-metre track. The examiner signalled the start of the test while standing by the participants’ side, carefully observing and informing about the remaining time after each minute elapsed (including the last 15 s) before the final stop sign.

The test–retest reliability of isometric strength and physical performance assessments within the Kosovo population has been previously demonstrated [25].

### Self-perceived health, lifestyle behaviour, and socio-economic status

Participants’ health status was assessed using the 7-item Physical Activity Readiness Questionnaire (PAR-Q) [26]. The WHO STEPS instrument was used to collect and analyse self-perceived health status, comorbidities and socioeconomic status (including the living environment, education level, marital and financial status, and behaviours such as smoking and alcohol consumption) [27]. The Mini Nutritional Assessment (MNA) questionnaire (long form) was used to assess participants' nutritional status, as an 18-item instrument consisting of anthropometric characteristics, dietary intake, general lifestyle assessment, and self-assessment of health and nutritional status [28]. Medication use and adherence were assessed using the Brief Medication Questionnaire [29]. The Physical Activity Scale for the Elderly (PASE) as a tool consisting of three components (leisure time, household, and work-related activities) was used to assess the physical activity level of the participants [30].

### Vitamin D intake and vitamin D knowledge

Vitamin D intake (µg/day) was assessed using the Vitamin D Estimation Only-Food Frequency Questionnaire (VIDEO-FFQ) [31]. This questionnaire is based on an assessment of food frequency, including the food products considered to be sources of vitamin D, and is considered to be an excellent tool for assessing vitamin D intake in populations without vitamin D data in food composition tables [32]. The validity of the VIDEO-FFQ has been demonstrated in a Croatian population [32], a neighbouring country of Kosovo with similar population characteristics (also being part of the former Yugoslavia until 1991). Subjects were asked to report the frequency of consumption of the foods in the portion sizes specified in the VIDEO-FFQ questionnaire. The calculation of vitamin D intake was made based on the information gathered from this questionnaire, which included details of food products, portion sizes, frequency of consumption , and the vitamin D content corresponding to each portion size.

In addition, to provide in-depth insights into the cognitive and behavioural patterns of the studied population regarding vitamin D in their daily routines, the participants’ knowledge, attitudes and practices (KAP) regarding vitamin D were assessed using the D-KAP-38 questionnaire. This instrument, was designed to assess a range of vitamin D-related aspects, including knowledge, attitudes, and habitual practices [33]. The assessment process was conducted using a four-point scoring system divided into the following categories: (1) general knowledge, including non-nutritional questions about vitamin D, such as those about sun exposure and sunscreen use; (2) nutritional knowledge, including questions about foods containing vitamin D; (3) attitudes, including questions about the participants’ attitudes towards different aspects of vitamin D-related information, such as the financial barriers posed by expensive vitamin D supplements; and (4) vitamin D-related behaviours assessing practices that potentially influence their vitamin D status, such as the regular use of sunscreen.

### Statistical analysis

Data analyses were performed using the SPSS 27 Windows statistical package (SPSS, Inc, Chicago, IL, USA), with the significance level set at *p* < 0.05. Descriptive statistics (mean and standard deviation for continuous variables, frequencies for categorical variables) were used as the means to describe participants’ general characteristics, including anthropometric characteristics, body composition, isometric strength and physical performance, physical activity and sedentary behaviour, self-perceived health status, comorbidities, medication use, and nutritional and socioeconomic status. According to the central limit theorem, we considered the sample size to be large enough to approximate a normal distribution for continuous variables [34]. Independent *t* tests were used to calculate differences between groups for continuous variables and χ2 tests were used for categorical variables. Effect sizes were calculated to estimate the magnitude of the effect. Cohen’s d was used for continuous variables, with effect sizes classified as small (0.2), medium (0.5) and large (0.8) [35], and Cramer’s V was used for categorical variables, with effect sizes classified as small (0.1), medium (0.3) and large (0.5) [36]. The association between possible determinants of serum 25(OH)D levels (nmol/L) and physical performance tests was analysed using a linear multiple regression model following the enter method. Linearity was assessed using partial regression plots and a plot of studentized residuals against the predicted values. The independence of residuals was assessed by Durbin–Watson statistics, while homoscedasticity was assessed by a visual inspection of plots of studentized residuals against the unstandardized predicted values. Finally, a Q–Q plot was used to assess the normality assumption [37].

## Results

### Participant characteristics

A total of 297 individuals aged ≥ 40 years participated in this study, about half of whom (138 or 46.5%) were recalled from a previous study [17, 18]. The characteristics of this study’s participants are summarised in Table 1. Of the 297 participants, 54.5% (*n* = 162) were female and 45.5% (*n* = 135) were male. Male participants were significantly older, taller and heavier, (*p* < 0.001), with higher lean muscle mass, body cell mass (*p* < 0.001) and phase angle (*p* = 0.002), but lower BMI (*p* = 0.001), total body fat mass and percentage (*p* < 0.001) than female participants. Interestingly, women were observed to have a larger mid-arm circumference (*p* = 0.017) than their male counterparts, whereas calf circumference was not significantly different between the sexes (*p* = 0.297).

**Table 1:** Anthropometric, physical performance, lifestyle and sociodemographic characteristics

Variable	Total [*n* = 297]	Women [*n* = 162]	Men [*n* = 135]	*p* value
Sex [%]	100	54.5	45.5	
Age [years]	65.3 ± 10.2	63.3 ± 10.2	67.6 ± 9.7	< *0.001*
Height [m]	1.64 ± 0.10	1.58 ± 0.07	1.71 ± 0.08	< *0.001*
Body mass [kg]	79.7 ± 14.3	76.3 ± 12.6	83.8 ± 15.1	< *0.001*
BMI [kg/m^2^]	29.7 ± 4.8	30.6 ± 4.9	28.7 ± 4.6	*0.001*
Whole body fat mass [kg]	28.9 ± 9.6	32.6 ± 9.1	24.5 ± 8.4	< *0.001*
Whole body fat percentage [%]	36.1 ± 9.4	42.2 ± 7.0	28.9 ± 6.3	< *0.001*
Lean muscle mass [kg]	50.8 ± 11.2	43.7 ± 6.6	59.2 ± 9.7	< *0.001*
Body cell mass [kg]	31.4 ± 6.4	27.3 ± 3.8	36.3 ± 5.1	< *0.001*
Phase angle [°]	5.2 ± 0.9	5.1 ± 0.8	5.4 ± 1.0	*0.002*
Mid-arm circumference [cm]	29.3 ± 3.6	29.8 ± 3.7	28.8 ± 3.4	*0.017*
Calf circumference [cm]	36.1 ± 4.8	36.3 ± 5.3	35.8 ± 4.1	*0.297*
Hand grip strength [kg]	31.7 ± 11.7	24.6 ± 6.7	40.3 ± 10.6	< *0.001*
Gait speed usual [m/s]	1.27 ± 0.21	1.24 ± 0.22	1.30 ± 0.20	*0.011*
Gait speed fast [m/s]	1.63 ± 0.33	1.55 ± 0.32	1.71 ± 0.33	< *0.001*
Timed up and go test [s]	7.18 ± 2.04	7.56 ± 2.20	6.72 ± 1.74	< *0.001*
30-s arm curl test [repetitions]	18 ± 4	18 ± 4	18 ± 4	*0.467*
30-s chair stand test [repetitions]	11 ± 3	11 ± 3	12 ± 3	*0.049*
6-min walking test [m]	470 ± 114	446 ± 115	498 ± 106	< *0.001*
PASE Total Activity Score [-]	100.4 ± 55.2	93.8 ± 48.4	108.4 ± 61.5	*0.025*
Mini nutritional status [-]	26 ± 2	26 ± 2	26 ± 2	*0.905*
Malnourished [yes/risk/no, %]	0.0/12.2/87.8	0.0/13.0/87.0	0.0/11.1/88.9	*0.612*
BMI categories [underweight/normal weight/overweight/obesity, %]	0.7/15.8/40.4/43.1	0.6/17.7/35.8/51.9	0.7/20.7/45.9/32.6	*0.007*
Smoking status [smoker/quit smoking/nonsmoker, %]	21.5/11.8/66.7	20.4/6.8/72.8	23.0/17.8/59.3	*0.007*
Self-perceived health condition [good/not good, %]	44.1/55.9	35.8/64.2	54.1/45.9	*0.002*
Self-declared chronic disease [yes/no, %]	68.4/31.6	76.5/23.5	58.5/41.5	*0.001*
Intake of medication [yes/no, %]	71.0/29.0	78.4/21.6	62.2/37.8	*0.002*
Number of medications [-]	2.1 ± 1.8	2.4 ± 1.8	1.7 ± 1.7	*0.003*
Education [no formal/1–8 years/ > 8 years, %]	2.0/32.7/65.3	3.1/42.6/54.3	0.7/20.7/78.5	< *0.001*
Marital status [single/partnership/married/widowed/divorced, %]	3.7/0/70.2/26.1/0	5.0/0.0/59.0/36.0/0.0	2.2/0.0/83.6/14.2/0.0	< *0.001*
Financial condition [enough to cover the month/not enough, %]	91.2/8.8	81.5/10.5	93.3/6.7	*0.245*

Data are expressed as means ± standard deviations or absolute numbers (percentages); Abbreviations: *BMI* body mass index. Independent *t* test was used for metric variables to determine differences between female and male participants, whereas Chi^2^ test was used for categorical variables; *p* < 0.05 was considered statistically significant

As expected, there were significant differences in HGS and physical performance tests, with male participants showing stronger HGS (*p* < 0.001), and better performance in TUG, 6MWT (*p* < 0.001), CS (*p* = 0.049) and gait speed at usual (*p* = 0.011) and fast pace (*p* < 0.001). Only AC performed with different weights was similar between men and women (*p* = 0.467).

In addition, male participants had a better PASE total activity score (*p* = 0.025), while nutritional status was similar in both sex groups. Significant differences were also observed within BMI categories, smoking status (both *p* = 0.007), self-perceived health status (*p* = 0.002) and chronic diseases (*p* = 0.001), and socioeconomic parameters such as educational level, and marital status (*p* < 0.001) but not self-declared financial status (*p* = 0.245). Female participants reported a higher percentage of medication intake (*p* = 0.002) and a higher number of consumed medications (*p* = 0.003).

### Vitamin D levels and the prevalence of vitamin D deficiency

As presented in Table 2, serum 25(OH)D levels were 56.6 ± 28.0 nmol/L in the total population (56.4 ± 31.6 nmol/L in female and 56.9 ± 23.0 nmol/L in male participants) with values ranging from 11.4 to 252.1 nmol/L. According to vitamin D categories, 45.7% of participants were found to be vitamin D deficient (< 50 nmol/L), of which 14.7% were severely deficient (< 30 nmol/L). Dietary vitamin D intake was reported to be as low as 1.89 ± 0.67 µg/d, and 75.8% of participants did not take any additional vitamin D supplements.

**Table 2:** Vitamin D status, intake and supplementation

Variable	Total [*n* = 297]	Women [*n* = 162]	Men [*n* = 135]	*p* value	Cohen’s d /Cramer’s V
Vitamin D level (nmol/L)	56.6 ± 28.0	56.4 ± 31.6	56.9 ± 23.0	*0.877*	*0.018*^*S*^
Vitamin D categories [< 29.9 nmol/L; 30.0–49.9 nmol/L; > 50.0 nmol/L, %]	14.7/32.8/52.6	18.9/28.9/52.2	9.7/37.3/53.0	*0.057*	*0.139*^*S*^
Vitamin D intake [µg/day]	1.89 ± 0.67	1.85 ± 0.67	1.94 ± 0.66	*0.255*	*0.135*^*S*^
Vitamin D supplementation [no/vitamin D only/vitamin D combination supplements/other, %]	75.8/8.1/4.4/11.8	67.3/12.3/6.2/14.2	85.9/3.0/2.2/8.9	*0.001*	*0.231*^*M*^

Data are expressed as means ± standard deviations or absolute numbers (percentages); Independent *t* tests were used for metric variables to determine differences between female and male participants, whereas Chi^2^ tests were used for categorical variables; *p* < 0.05 was considered statistically significant; effect sizes for continuous variables: Cohen’s d small (S), medium (M), large (L); effect sizes for categorical variables: Cramer’s V small (S), medium (M), large (L)

There were no significant differences in vitamin D levels (*p* = 0.057) or intake (*p* = 0.255) between men and women. However, vitamin D supplementation alone or in combination with other supplements was reported more frequently by women (*p* = 0.001). As shown in Supplementary Table 1, there were no significant differences between the sexes in dietary knowledge about vitamin D (*p* = 0.658). However, female participants had higher scores for general knowledge (non-nutritional questions about vitamin D, such as questions about sun exposure and sunscreen use, *p* < 0.001) and attitudes (towards various aspects of vitamin D-related information, such as the financial barriers posed by expensive vitamin D supplements, *p* = 0.013), but lower scores for vitamin D practice (vitamin D-related behaviours, evaluation of practices that potentially affect their vitamin D status, such as regular use of sunscreen, *p* < 0.001).

### Vitamin D levels and associated factors

As sex is an important determinant of physical performance, sex-specific descriptive data on anthropometrics, body composition and functional performance are presented separately for males and females in Table 3. In male participants, vitamin D was positively correlated with gait speed (*r* = 0.256, *p* = 0.003), fast gait speed (*r* = 0.203, *p* = 0.019), armcurl test (*r* = 0.178, p = 0.039), chair stand test (*r* = 0.202, *p* = 0.020) and 6MWT (*r* = 0.239, *p* = 0.005). As a result, men with low vitamin D levels (< 50 nmol/L) showed poorer performance on the usual and fast gait speed tests (*p* = 0.005 and 0.004 respectively), as well as the 30-s arm curl (*p* = 0.045) and 30-s chair stand (*p* = 0.010) tests, compared with men with adequate vitamin D levels (≥ 50 nmol/L). Therefore, effect sizes for functional tests were shown to be of medium to large size. Interestingly, we did not find any correlations between these variables and vitamin D levels in women. Nevertheless, the results for the 30-s chair stand test were worse in women with a deficient vitamin D status (*p* = 0.003).

**Table 3:** Anthropometrics, body composition and functional performance of the male and female participants by vitamin D deficiency status

	Men					Women				
Variable	Total (*n* = 135)	Deficient* (*n* = 64)	Sufficient** (*n* = 71)	p value	Cohen’s d /Cramer’s V	Total (*n* = 162)	Deficient* (*n* = 79)	Sufficient** (*n* = 83)	*p* value	Cohen’s d /Cramer’s V
Age [years]	67.6 ± 9.7	67.4 ± 11.2	67.8 ± 8.2	*0.808*	*0.041*^*S*^	63.3 ± 10.2	63.8 ± 11.0	62.8 ± 9.5	*0.514*	*0.097*^*S*^
Height [m]	1.71 ± 0.08	1.71 ± 0.08	1.71 ± 0.08	*0.950*	*0.000*^*S*^	1.58 ± 0.07	1.58 ± 0.07	1.58 ± 0.07	*0.986*	*0.000*^*S*^
Body mass [kg]	83.8 ± 15.1	84.9 ± 16.6	82.8 ± 13.6	*0.409*	*0.138*^*S*^	76.3 ± 12.6	75.8 ± 14.1	76.8 ± 11.0	*0.625*	*0.079*^*S*^
BMI [kg/m^2^]	28.7 ± 4.6	29.1 ± 5.1	28.3 ± 4.0	*0.343*	*0.166*^*S*^	30.5 ± 4.9	30.32 ± 5.4	30.76 ± 4.3	*0.575*	*0.898*^*L*^
Whole body fat mass [kg]	24.5 ± 8.4	24.9 ± 9.2	24.2 ± 7.6	*0.626*	*0.083*^*S*^	32.6 ± 9.1	32.5 ± 10.2	32.7 ± 8.0	*0.890*	*0.022*^*S*^
Whole body fat percentage [%]	28.9 ± 6.3	28.9 ± 6.5	28.9 ± 6.1	*0.960*	*0.000*^*S*^	42.2 ± 7.0	42.1 ± 7.8	42.2 ± 6.3	*0.971*	*0.014*^*S*^
Lean Muscle Mass [kg]	59.2 ± 9.7	59.9 ± 10.4	58.6 ± 9.2	*0.442*	*0.132*^*S*^	43.7 ± 6.6	43.4 ± 7.4	44.1 ± 5.9	*0.522*	*0.104*^*S*^
Body cell mass [kg]	36.3 ± 5.1	36.7 ± 5.7	36.0 ± 4.7	*0.380*	*0.134*^*S*^	27.3 ± 3.8	27.1 ± 4.2	27.4 ± 3.4	*0.731*	*0.079*^*S*^
Phase angle [°]	5.4 ± 1.0	5.4 ± 1.1	5.4 ± 1.0	*0.779*	*0.000*^*S*^	5.0 ± 0.8	5.0 ± 0.8	5.1 ± 0.8	*0.266*	*0.125*^*S*^
Hand grip strength [kg]	40.3 ± 10.6	41.2 ± 11.6	39.5 ± 9.6	*0.328*	*0.160*^*S*^	24.6 ± 6.7	24.7 ± 6.7	24.5 ± 6.8	*0.806*	*0.030*^*S*^
Gait speed usual [m/s]	1.30 ± 0.20	1.25 ± 0.21	1.35 ± 0.18	*0.005*	*0.511*^*L*^	1.24 ± 0.22	1.22 ± 0.21	1.26 ± 0.22	*0.227*	*0.186*^*S*^
Gait speed fast [m/s]	1.71 ± 0.33	1.63 ± 0.32	1.79 ± 0.32	*0.004*	*0.500*^*M*^	1.55 ± 0.32	1.54 ± 0.33	1.56 ± 0.31	*0.690*	*0.062*^*S*^
Timed up and go test [s]	6.72 ± 1.74	6.94 ± 1.99	6.53 ± 1.46	*0.162*	*0.235*^*M*^	7.56 ± 2.20	7.85 ± 2.56	7.29 ± 1.76	*0.106*	*0.255*^*M*^
30-s arm curl test [repetitions]	18 ± 4	17 ± 4	19 ± 4	*0.045*	*0.500*^*M*^	18 ± 4	18 ± 4	18 ± 4	*0.832*	*0.000*^*S*^
30-s chair stand test [repetitions]	12 ± 3	11 ± 3	12 ± 3	*0.010*	*0.333*^*M*^	11 ± 3	10 ± 3	11 ± 3	*0.003*	*0.333*^*M*^
6-min walking test [m]	498 ± 106	480 ± 90	514 ± 117	*0.060*	*0.326*^*M*^	446 ± 115	440 ± 103	452 ± 126	*0.504*	*0.104*^*S*^

Data are expressed as means ± standard deviations or absolute numbers (percentages); Abbreviations: *BMI* (body mass index). Independent t test was used for metric variables to determine differences between individuals with vitamin D sufficiency and deficiency, whereas Chi^2^ test was used for categorical variables; *p* < 0.05 was considered statistically significant; effect sizes for continuous variables: Cohen’s d small (S), medium (M), large (L); effect sizes for categorical variables: Cramer’s V small (S), medium (M), large (L). * Deficient: Vitamin D < 50 nmol/L; ** sufficient: Vitamin D ≥ 50 nmol/L

When analysing various lifestyle factors (Table 4), we did not find any differences between the groups with low and sufficiently high vitamin D levels, except for small differences in the smoking status of women (*p* = 0.023).

**Table 4:** Lifestyle, health and socioeconomic factors in male and female participants by vitamin D deficiency status

	Men					Women				
Variable	Total (*n* = 135)	Deficient* (*n* = 64)	Sufficient** (*n* = 71)	*p* value	Cohen’s d /Cramer’s V	Total (*n* = 162)	Deficient* (*n* = 79)	Sufficient** (*n* = 83)	p value	Cohen’s d /Cramer’s V
PASE Total Activity Score [-]	108.4 ± 61.5	110.1 ± 64.2	106.9 ± 59.4	*0.767*	*0.052*^*S*^	93.8 ± 48.4	91.7 ± 48.7	95.7 ± 48.4	*0.607*	*0.082*^*S*^
Mini nutritional status [-]	26 ± 2	26 ± 2	26 ± 2	*0.944*	*0.000*^*S*^	26 ± 2	26 ± 2	26 ± 2	*0.132*	*0.000*^*S*^
Smoking status [smoker/quit smoking/nonsmoker, %]	23.0/17.8/59.3	21.9/10.9/67.2	23.9/23.9/52.1	*0.102*	*0.184*^*M*^	20.4/6.8/72.8	22.8/1.3/75.9	18.1/12.0/69.9	*0.023*	*0.216*^*M*^
Self-perceived health condition [good/not good, %]	45.9/54.1	43.8/56.2	47.9/52.1	*0.630*	*0.041*^*S*^	64.2/35.8	64.6/35.4	63.9/36.1	*0.926*	*0.008*^*S*^
Self-declared chronic disease [yes/no, %]	41.5/58.5	39.1/60.9	43.7/56.3	*0.588*	*0.046*^*S*^	23.5/76.5	27.8/72.2	19.3/80.7	*0.198*	*0.101*^*M*^
Intake of medication [yes/no, %]	32.6/67.4	34.4/65.6	31.0/69.0	*0.675*	*0.037*^*S*^	17.4/82.6	19.0/81.0	15.9/84.1	*0.600*	*0.042*^*S*^
Education [no formal/1–8 years/ > 8 years, %]	0.7/20.7/78.5	0.0/28.1/71.9	1.4/14.1/84.5	*0.091*	*0.188*^*M*^	3.1/42.6/54.3	2.5/48.1/49.4	3.6/37.3/59.0	*0.377*	*0.110*^*M*^
Marital status [single/partnership/married/widowed, divorced, %]	2.2/0.0/83.0/14.1/0.7	1.6/84.4/14.1/0.0	2.8/81.7/14.1/1.4	*0.760*	*0.093*^*S*^	4.9/1.9/56.8/35.8/0.6	6.3/1.3/59.5/32.9/0.0	3.6/2.4/54.2/38.6/1.2	*0.663*	*0.122*^*M*^
Financial condition [enough to cover the month/not enough, %]	93.3/6.7	93.8/6.3	93.0/7.0	*0.854*	*0.015*^*S*^	89.5/10.5	92.4/7.6	86.7/13.3	*0.240*	*0.092*^*S*^

Data are expressed as means ± standard deviations or absolute numbers (percentages); Abbreviations: *BMI* (body mass index), PASE (physical activity scale for the elderly). Independent *t* tests were used for metric variables to determine differences between persons with vitamin d sufficiency and deficiency, whereas Chi^2^ tests were used for categorical variables; *p* < 0.05 was considered statistically significant; effect sizes for continuous variables: Cohen’s d small (S), medium (M), large (L); effect sizes for categorical variables: Cramer’s V small (S), medium (M), large (L). * Deficient: Vitamin D < 50 nmol/L; ** sufficient: Vitamin D ≥ 50 nmol/L

Vitamin D-related knowledge, attitudes and practices are shown in Supplementary Table 2. Significantly lower scores for vitamin D attitudes and practices were found among vitamin D-deficient women (*p* = 0.011 and *p* = 0.022, respectively). As expected, the proportion of females with vitamin D intake was higher in the vitamin D sufficient group (*p* < 0.001). Interestingly, no differences in these variables were found between men with low and sufficiently high levels of vitamin D.

### Factors associated with vitamin D levels and other covariates

A multiple regression was performed to potentially predict vitamin D status from several other covariates (sex, age, vitamin D intake, general knowledge, nutritional knowledge, attitude, practice and supplementation). Vitamin D supplementation (*p* = 0.009) was the only variable that added statistically significantly to the overall model F(8, 284) = 1.430, *p* = 0.183. There was independence of residuals, as assessed by a Durbin–Watson statistic of 1.915 and homoscedasticity, as assessed by visual inspection of a plot of studentized residuals versus unstandardised predicted values. The *R*^2^ for the overall model was 3.9%, with an adjusted *R*^2^ of 1.2%.

Multiple regression analyses were also run to predict the physical performance parameters CS, gait speed (slow pace) and AC, from vitamin D levels and other potential confounders (sex, age, body mass, nutritional status, physical activity score, self-perceived health condition, medication intake, vitamin D intake, general vitamin D knowledge, nutritional knowledge, practice and supplementation). The physical performance parameters were selected on the basis of the significant differences observed between the low and normal vitamin D level groups (Table 3).

For CS, there was an independence of residuals, as assessed by a Durbin–Watson statistic of 1.549. Homoscedasticity was checked by visual inspection of a plot of studentized residuals against unstandardised predicted values. Collinearity statistics show values above 0.446, making multicollinearity rather unlikely. The *R*^2^ for the overall model was 24.7%, with an adjusted *R*^2^ of 20.8%. The overall model significantly predicted CS performance, F(14,274) = 6.406, *p* < 0.001, with only age (*p* = 0.013), medication intake (*p* = 0,003) and vitamin D level (*p* = 0.008) adding statistically significant to the prediction. Regression coefficients and standard errors are shown in Table 5.

**Table 5:** Factors associated with chair stand test performance

Chair stand	B	95% CI	SE B	β	*R*^2^	ΔR^2^	
		LL	UL				
*Model*						0.247	0.208***
Constant	3.433	– 3.434	10.301	3.488			
Sex	0.755	– 0.035	1.545	0.401	0.124		
Age	– 0.055*	– 0.099	– 0.012	0.022	– 0.184		
Body mass	– 0.016	– 0.041	0.008	0.013	– 0.077		
Mini nutritional status	0.163	– 0.013	0.340	0.089	0.110		
PASE Total Activity Score	0.000	– 0.008	0.007	0.004	– 0.008		
Health condition	– 0.056	– 0.876	0.765	0.417	– 0.009		
Medication intake	– 1.491**	– 2.478	– 0.505	0.501	– 0.211		
Vitamin D level	0.039**	0.010	0.068	0.015	0.143		
Vitamin D intake	0.128	– 0.381	0.637	0.259	0.028		
Vitamin D general knowledge	0.038	– 0.100	0.175	0.070	0.042		
Vitamin D nutritional knowledge	0.066	– 0.155	0.287	0.112	0.032		
Vitamin D attitude	0.090	– 0.026	0.205	0.059	0.116		
Vitamin D practice	0.098	– 0.017	0.212	0.058	0.097		
Vitamin D supplementation	– 0.078	– 0.413	0.256	0.170	– 0.026		

Note. Model = “Enter” method in *SPSS* Statistics, *B*  unstandardized regression coefficient; *CI*  confidence interval, *LL*  lower limit, *UL*  upper limit, *SE B*  standard error of the coefficient, *β*  standardized coefficient, *R*^2^  coefficient of determination; Δ*R*^2^  adjusted *R*^2^. *p < 0.05, **p < 0.01, ***p < 0.001

For the usual gait speed assessment, only sex (*p* = 0.010) and vitamin D practice (behaviours associated with vitamin D, assessment of practices that potentially influence their vitamin D status; *p* = 0.045) were the variables that added statistically significant predictive value (F(14, 276) = 2.161, *p* = 0.009). This gave an R^2^ for the overall model of 9.9% and an adjusted *R*^2^ of 5.3% (data not shown).

For AC performance, the regression model did not even reach significance F(14,275) = 1.601, *p* = 0.078 with an *R*^2^ of 7.5% and an adjusted R^2^ of 2.8% for the overall model (Durbin–Watson statistics = 1.700). The only variable that showed a significant association with AC performance was age (*p* = 0.023).

## Discussion

This study investigated vitamin D status in the adult population of Kosovo aged over 40 years and compared these data with muscle characteristics and various sociod-demographic and socio-economic factors. The main findings meet the objectives by showing that vitamin D deficiency is highly prevalent among the Kosovar population. During winter (December–February), 47.8% of the female and 47.0% of the male population had serum 25(OH)D concentrations below 50 nmol/L, which is considered to be vitamin D deficiency. There was no significant association between 25(OH)D concentration and age. Not surprisingly, vitamin D supplementation was the only variable that added statistical significance (*p* < 0.05) to the prediction of vitamin D status (2.1%). Similarly, for upper body endurance strength (AC) and gait speed (from the physical performance assessments), age and vitamin D practice and attitude were the variables that contributed to their specific models (3.5% and 7.5%, respectively). In particular, the lower body strength endurance test (CS) stood out among the physical performance tests, with variables with a significant contribution (*p* < 0.005) explaining 24.7% of its variance from age, vitamin D practice, vitamin D attitude, sex and vitamin D status.

A wide range of vitamin D deficiency has been reported in adult populations worldwide, regardless of geographical or population specific characteristics. In Europe, it has been identified as a pandemic, warranting increased public health attention [2]. The European Calcified Tissues Society (ECTS) reported prevalence ranging from 0.4 to 8.4% (threshold set at < 30 nmol/L) and 6.6%–33.6% (< 50 nmol/L) in Northern Europe, 4.6–30.7% (< 30 nmol/L) and 27.2%–61.4% (< 50 nmol/L) in Western Europe, and no standardised data were available for adults in Southern and Eastern Europe [38]. Cashman and colleagues [2] suggested a deficiency prevalence of 13.0% (< 30 nmol/L) to 41.4% (< 50 nmol/L) in Europeans of all ages. However, it has been shown [39] that studies providing plausible data on vitamin D status data are lacking for almost two-thirds of low- and middle-income countries (LMICs). Some of these countries reported very high prevalence of vitamin D deficiency, such as 80.1% and 99.6% (< 50 nmol/L and < 75 nmol/L, respectively) in Mongolian women (46.8°N, winter season) [40], 53.5% in Pakistani men and women combined (< 50 nmol/L, 34.0°N, all seasons) [41], 54.1% in Iranian women and 46% in men (< 50 nmol/L, 32.4°N, [42]), and 40.8% in Russian men and women (< 50 nmol/L, 61.5°N, all seasons) [43].

There appears to be  a general lack of data on vitamin D status in the adult population of Kosovo. A year-round study from Bosnia and Herzegovina (adults aged > 18 years) showed a prevalence of vitamin D deficiency (< 50 nmol/l) of 60.6% and insufficiency (50–75 nmol/L) of 21.4% in the population studied [44]. Another country in the region reporting winter (January–February) deficiency prevalence in adults is Bulgaria [45], with 21.3% in the < 25 nmol/L range (26.9% in women and 15.1% in men) and 54.5% in the 25.00–49.99 nmol/L range (53.7% in womene and 55.3% in ). Even the southern Balkan country of Greece (39.1°N) described a deficiency of 64.8% following the < 50 nmol/L threshold [46]. However, the highest regional prevalence of vitamin D deficiency was reported in a study from neighbouring Serbia, whereh vitamin D deficiency in young adult women was 90% (< 50 nmol/l) and 70% (< 30 nmol/l) sampling late during winter [47].

With regard to the possible sex differences, this study aligns with the majority of studies describing no sex-specific potential to develop vitamin D deficiency [2, 48, 49]. However, female participants scored significantly higher on general knowledge and attitudes about vitamin D and vitamin D supplementation. Similar results were previously reported by Amiri and colleagues [33]. This may be due to their propensity to receive more health information from the media and more advice from health professionals to take vitamin D and calcium supplements to prevent osteoporosis. Meanwhile, lower scores in vitamin D-related practices might be influenced by several socio-environmental barriers related to cultural perceptions and behaviours [33, 50]. Smoking was also found to be a significant contributing factor to vitamin D deficiency. The percentage of subjects who smoked was higher in the deficient group (22.8%) than inthe other group (18.1%), while even those who had stopped smoking were more likely to be in the non-deficient group (12.0% vs. only 1.3% in the deficient group). Some previous studies have investigated and described possible associations between smoking and vitamin D deficiency in women [51, 52], emphasising the need for further investigation.

The male group with vitamin D deficiency was characterised by significantly lower physical performance parameters, including gait speed (both normal and fast pace), AC and CS. Such findings highlight the potential influence of vitamin D deficiency on walking speed which has already been confirmed in a meta-analysis [53]. Furthermore, the influence of vitamin D deficiency on both upper and lower body strength (endurance) in our male participants is another important factor to consider. Although similar findings have been shown previously [10, 54], the association with male sex only, even with smaller effect sizes, is a novelty. This could be explained by the possible effects of 25(OH)D on the male reproductive system, including testosterone production [55], which contributes to neuromuscular development by having a significant anabolic effect on muscle tissue [56]. On the other hand, alongside evidence that higher levels of overweight and obesity may signal impaired muscle function [57, 58], the lack of association in female participants needs further consideration. Although, this study did not causally test this hypothesis, it may be that the higher levels of overweight and obesity, especially in female participants, may have interacted with cultural aspects that differ between males and females, potentially obscuring the findings in other parameters except for the CS performance.

The results from this study showed that lower vitamin D status, combined with lower scores on vitamin D-related practices, as well as being older and of female, explained about a quarter (24.7%) of the variance in lower body strength endurance (via CS). However, for the other two physical performance parameters (AC and slow gait speed), fewer influencing variables were identifierd, with neither sex nor vitamin D practice (for gait speed) and only age (for AC) contributing to their specific regression models. When analysing the potential covariates influencing vitamin D status, vitamin D supplementation was the only variable that added significantly to the prediction, but accounted for only 3.9% of the variance. In addition, our data analysis showed that 87.6% of people did not take vitamin D supplements. This is particularly notable given that the serum was collected in winter and that our subjects were characterised by a very low daily dietary intake of vitamin D (1.89 ± 0.67 µg/day). However, the most recent data from the European Food Saferty Agency (EFSA), based on European dietary surveys, show that vitamin D intake (from food sources) ranges from 2.48 to 4.34 µg/day in adult and older adult men and from 1.84 to 3.53 µg/day in women. In addition, the EFSA recommends an adequate intake (AI) of 15 µg/day for adults [1]. The low dietary intake of vitamin D among the Kosovar population compared to the recommendations highlights the urgent need for interventions, including even food fortification strategies as a potential way to improve the prevalence of vitamin D deficiency in Kosovo. Interestingly, the significantly lower education of female participants compared to male participants did not prevent them from having higher general knowledge about vitamin D, resulting in significantly higher supplementation rates.

A final point of concern observed in this study was the high levels of overweight and obesity among the study participants (40.4% and 43.1% in the total population, 35.8% and 51.9% in females, 45.9% and 32.6% in males) highlighting this as a growing public health concern. Similarly high rates were reported in our previous publication [17, 18]. Considering the fact that vitamin D insufficiency and deficiency in people with obesity are thought to be a result of the increased distribution volume due to deposition in body fat compartments [59], the high percentage of overweight and obesity in our study population may explain the higher levels of vitamin D insufficiency and deficiency.

The main strengths of this study are the novelty of its data in an understudied population setting, providing the first data on vitamin D status and intake in Kosovo, which could be a starting point for the whole Balkan region. Furthermore, the comprehensive in-depth examination of the relationship between vitamin D status, muscle function and performance, health and basic socioeconomic data provided the first insights into the complex relationship between vitamin D, biological functions, and behavioural and financial aspects.

Although this study was carried out with the outmost care, there are certain limitations. Notwithstanding the merits of cross-sectional studies in this area, there may be a need for larger epidemiological studies, especially in under-researched populations, using more objective methods (rather than questionnaires). In addition, the very high prevalence of overweight and obesity in the study population may have confounded the outcome measures.

## Conclusion

This study showed that vitamin D deficiency was prevalent among community-dwelling adults in Kosovo and represents a daunting prospect for health-related conditions. These data indicate that this deficiency should be considered as a serious public health problem, implying an urgent need for the development of both short- and long-term prevention strategies, focusing on non-pharmacological strategies such as education and possibly food fortification. Furthermore, the findings of associations of serum 25(OH)D concentrations with measures of strength-related outcomes (such as CS) may facilitate the process of intervention development by targeting both health and lifestyle qualities.

### Supplementary Information

Below is the link to the electronic supplementary material.

Supplementary file1 (PDF 223 KB)

## Data Availability

The data presented in this study are available on request from the corresponding author.
