# Supplementary material for: Association between vitamin D status, physical performance, sex, and lifestyle factors: a cross-sectional study of community-dwelling Kosovar adults aged 40 years and older
Source: Eur J Nutr. 2024 Jan 9;63(3):821–34. doi: 10.1007/s00394-023-03303-9 (PMC10948476; doi:10.1007/s00394-023-03303-9)
Supplement: Supplementary file 1 — Supplementary file1 (PDF 223 KB) [file 394_2023_3303_MOESM1_ESM.pdf]

Online Supporting Information

Association between vitamin D status, physical performance, sex, and lifestyle factors: a cross-sectional study of community-dwelling Kosovar adults aged 40 years and older

Ermira Krasniqi[1,2,3], Arben Boshnjaku[2,3,5], Antigona Ukëhaxhaj[5,6], Karl-Heinz Wagner[1,4], Barbara Wessner[1,3] *

[1] Research Platform Active Ageing, University of Vienna, Josef-Holaubek-Platz 2, 1090 Vienna, Austria

[2] Vienna Doctoral School of Pharmaceutical, Nutritional and Sport Sciences (PhaNuSpo), Josef-Holaubek-Platz 2, University of Vienna, 1090 Vienna, Austria

[3] Centre for Sport Science and University Sports, University of Vienna, Auf der Schmelz 6, 1150 Vienna, Austria

[4] Department of Nutritional Sciences, University of Vienna, Josef-Holaubek-Platz 2, 1090 Vienna, Austria

[5] University "Fehmi Agani" in Gjakova, Ismail Qemali n.n. 50000, Gjakovë, Kosovo

[6] National Institute of Public Health of Kosovo, Centre for Laboratory Testing, Mother Teresa, n.n., Hospital District, 10000, Prishtina, Kosovo

*Corresponding author

Email addresses: barbara.wessner@univie.ac.at

Manuscript submitted to: European Journal of Nutrition

Supplementary Table 1. Vitamin D knowledge, attitude and practice

Variable	Total [n=297]	Women [n=162]	Men [n=135]	p value	Cohen's d /Cramer's V
Vitamin D general knowledge scores [-][a]	14.67 ± 3.44	15.31 ± 3.46	13.89 ± 3.26	*<0.001*	*0.422*[M]
Vitamin D nutritional knowledge scores [-][b]	4.57 ± 1.48	4.53 ± 1.57	4.61 ± 1.37	*0.658*	*0.054*[S]
Vitamin D attitude scores [-][c]	39.03 ± 3.92	39.54 ± 3.90	38.41 ± 3.87	*0.013*	*0.291*[S]
Vitamin D practice scores [-][d]	30.93 ± 3.07	30.23 ± 2.96	31.76 ± 3.01	*<0.001*	*0.513*[M]

Data are expressed as means ± standard deviations; Independent t tests were used for metric variables to determine differences between female and male participants; $p<0.05$ was considered statistically significant; effect sizes for continuous variables: Cohen's d small (S), medium (M), large (L); effect sizes for categorical variables: Cramer's V small (S), medium (M), large (L).

[a] nonnutritional inquiries about vitamin D, such as those about sun exposure and the use of sunscreen; [b] inquiries about foods containing vitamin D knowledge; [c] inquiries about the participants' attitudes towards different aspects of vitamin D-related information, like the financial obstacles posed by pricey vitamin D supplements; [d] behaviors associated with vitamin D, appraising practices that potentially influence their vitamin D status, for instance, the regular application of sunscreen

Supplementary Table 2. Vitamin D knowledge, attitude and practice in male and female participants by vitamin D deficiency status

	Men					Women				
**Variable**	**Total (n=135)**	**Deficient* (n=64)**	**Sufficient** (n=71)**	**p value**	**Cohen's d /Cramer's V**	**Total (n=162)**	**Deficient* (n=79)**	**Sufficient** (n=83)**	**p value**	**Cohen's d /Cramer's V**
Vitamin D intake [µg/day]	1.94 ± 0.66	1.85 ± 0.57	2.02 ± 0.73	*0.154*	*0.260*[M]	1.85 ± 0.67	1.84 ± 0.67	1.86 ± 0.67	*0.785*	*0.030*[S]
Vitamin D general knowledge scores [0-22] [a]	13.89 ± 3.26	14.00 ± 3.36	13.79 ± 3.18	*0.708*	*0.064*[S]	15.31 ± 3.46	14.87 ± 3.50	15.73 ± 3.38	*0.113*	*0.250*[M]
Vitamin D nutritional knowledge scores [0-10] [b]	4.61 ± 1.37	4.45 ± 1.31	4.75 ± 1.41	*0.214*	*0.220*[M]	4.53 ± 1.57	4.62 ± 1.44	4.44 ± 1.68	*0.479*	*0.115*[S]
Vitamin D attitude scores [12-60] [c]	38.41 ± 3.87	38.84 ± 4.12	38.03 ± 3.60	*0.223*	*0.209*[M]	39.54 ± 3.90	38.75 ± 3.55	40.30 ± 4.09	*0.011*	*0.405*[M]
Vitamin D practice scores [10-50] [d]	31.76 ± 3.01	31.25 ± 2.57	32.22 ± 3.30	*0.060*	*0.328*[M]	30.23 ± 2.96	29.68 ± 2.68	30.75 ± 3.13	*0.022*	*0.367*[M]
Vitamin D supplementation [no/vitamin D only/vitamin D combination supplements/other, %]	53.5/1.8/1.4/5.5	90.6/3.1/0.0/6.3	81.7/2.8/4.2/11.3	*0.264*	*0.171*[M]	67.3/12.3/6.2/14.2	82.3/5.1/0.0/12.7	53.0/19.3/12.0/15.7	*<0.001*	*0.365*[L]

Data are expressed as means ± standard deviations or absolute numbers (percentages). Independent t tests were used for metric variables to determine differences between persons with vitamin d sufficiency and deficiency, whereas Chi² tests were used for categorical variables; $p<0.05$ was considered statistically significant; effect sizes for continuous variables: Cohen's d small (S), medium (M), large (L); effect sizes for categorical variables: Cramer's V small (S), medium (M), large (L). * deficient: Vitamin D <50 nmol/L; ** sufficient: Vitamin D ≥50 nmol/L [a] nonnutritional inquiries about vitamin D, such as those about sun exposure and the use of sunscreen; [b] inquiries about foods containing vitamin D knowledge; [c] inquiries about the participants' attitudes towards different aspects of vitamin D-related information, like the financial obstacles posed by pricey vitamin D supplements; [d] behaviors associated with vitamin D, appraising practices that potentially influence their vitamin D status, for instance, the regular application of sunscreen
